# Genes: an Open Access Journal

**DOI:** 10.3390/genes101

**Published:** 2009-11-02

**Authors:** J. Peter W. Young

Genes have been in the scientific vocabulary for a hundred years. The term "gene" was proposed by the Danish plant scientist Wilhelm Johannsen in the first decade of the 20th century. For Johannsen, the gene remained an abstract concept, "free of any hypothesis" [1], but others were already pointing to chromosomes as the likely location of genes. The science of genetics was born at that time, and genes were rapidly connected with mutations, with patterns of inheritance, with development, with quantitative traits, with evolution and with biochemical pathways. All this was achieved without knowledge of the physical nature of genes, but this changed in mid-century with the discoveries of molecular biology. DNA was revealed as the genetic material, and the mechanisms were elucidated by which the information was encoded, and propagated, and linked to the phenotype. However, the concept of a "gene" did not become clearer. Quite the reverse, as the units of mutation, of recombination, of inheritance, of expression, of regulation, *etc.* did not necessarily coincide. 

Genes are so embedded in our intellectual DNA (to repatriate a fashionable idiom) that we tend to forget that we still have no agreed definition of the term [2]. When we say that there are fewer than 25,000 genes in the human genome [3], we are thinking of protein-coding sequences, but are the associated introns and regulatory sequences part of the gene? And what about splice variants, untranslated RNAs, epigenetic modifications? These are important aspects of the genetic material and currently hot research topics. Furthermore, genes still occupy a central place in our modern understanding of biology, and all the connections that preoccupied our predecessors a century ago are still lively issues. The explosion of genome data and of new techniques is generating new insights in human diversity, developmental biology, molecular evolution, systems biology and many other directions.

We may not know exactly what "genes" are, but I can tell you what *Genes* is. *Genes* is a new Open Access, online-only journal covering all gene-related biology: genes, genetics and genomics, and the science that they enable. 

A list of some relevant keywords will give you a flavour of the scope: DNA; RNA; chromosomes; reproduction; heredity; genealogy; interaction of multiple genes; genetic code; pseudogene; gene structure; gene expression; recombination and linkage; genetic mapping; inheritance; nature versus nurture; gene regulation; genetic change; population genetics; conservation genetics; phylogenomics; phylogenetics; cloning; genetically modified organisms; human genetics; comparative genomics; behavioural genetics; medical genetics; gene therapy; genome projects; personal genomics; copy number variation; public health genomics; genetic diversity; transcriptional profiling; microRNA analysis; mRNA analysis; analysis of noncoding and other RNAs. 

Why do we need a new journal in the area of genetics and genomics? It is true that there are many established journals that cover this field, but the field itself is expanding. The result is that good quality journals are receiving increasing numbers of submissions, resulting in slow processing times for reviewing and publication and a lottery in which perfectly sound papers can eventually be rejected. When you need the publication for your final report, degree, promotion or next grant application, such uncertainty can be stressful. Our aim for *Genes* is to reduce delays to a minimum and to provide a good experience for authors and readers alike. That is why *Genes* is an Open Access, online-only journal managed by a professional office in Switzerland. 

The advantage of Open Access is that everyone with internet access can read the articles at no cost – not just those at institutions that can afford the subscription. This increases the number of people who read and cite the work. Of course, nothing is free in this life, so the cost of publication is borne by the authors. The publication charges of MDPI journals are relatively modest, though, compared to many other Open Access journals, and certainly compared to the costs of carrying out the research. Even better, publication charges will be waived for all articles that are submitted to *Genes* before the end of 2010, so there is every incentive to submit your papers soon.

The first advantage of an online-only journal is speed of publication. *Genes* is a peer-reviewed publication: all manuscripts are evaluated by independent anonymous referees in the usual way, but we will ensure that this is done as speedily as possible. Once a manuscript is accepted, it can be prepared for on-line publication much faster than for a print journal. Furthermore, there is no need to wait until an "issue" is complete, so each article will be published as soon as it is ready. The result is that work gets published faster and cited sooner. A further advantage of online publication is that there is effectively no space limitation. There is no need to reject good papers because the journal is "full", and every reason to make available all the relevant data to support a study's findings.

I am happy to be supported in this venture by a strong Editorial Board of active scientists who cover the wide remit of the journal. It is their expertise and judgment that will ensure that the articles published in *Genes* will command respect and be worthy of attention. Though we aim to be rapid, the peer review standards will be as high as we have come to expect in this field. This journal is run by geneticists for geneticists. We are fortunate to have the backing of MDPI, an organisation with more than a decade of experience in online, Open Access science publishing, and dedicated editorial office staff who have a science background. 

*Genes* publishes reviews, research articles, communications and technical notes. In addition, manuscripts regarding research proposals and research ideas will be particularly welcomed. Special Issues are organised around a defined theme; they will often be edited by a member of the Editorial Board, but we welcome proposals from the whole genetics community. For example, a Special Issue may be devoted to selected papers presented at a relevant conference. Articles do not have to be in a Special Issue, however: independent submissions are welcome at any time, and accepted papers will be published as soon as they are ready.

We aim to provide a rapid and hassle-free publication route for authors, so that they will continue to come back to *Genes* with good manuscripts. If you have work to publish, why not try us? It is free until the end of 2010.
